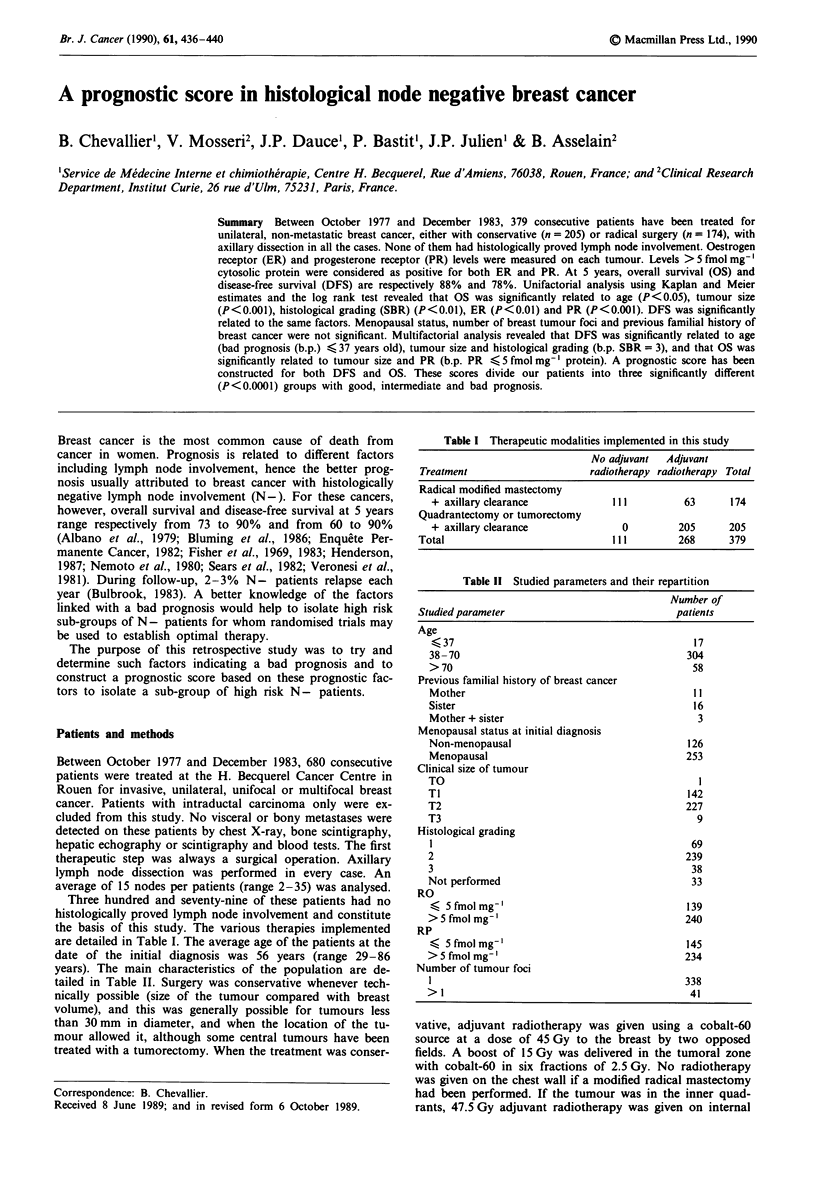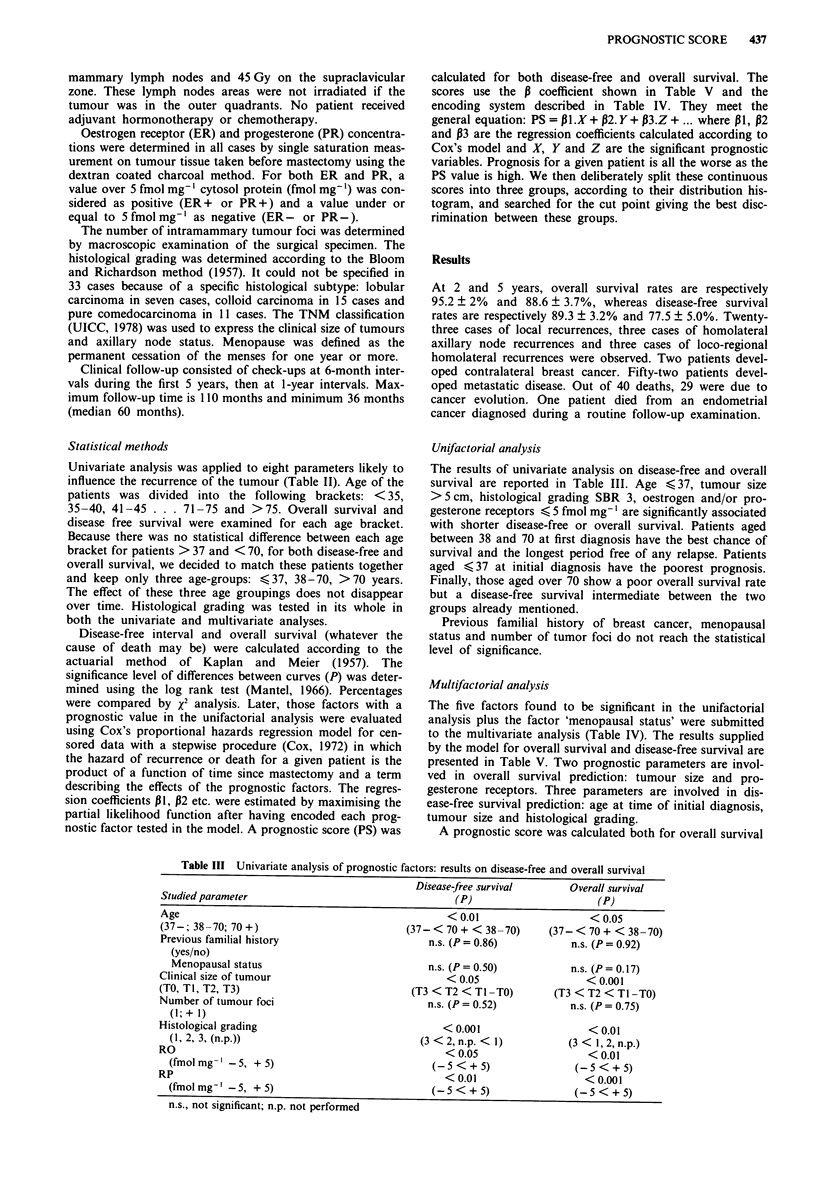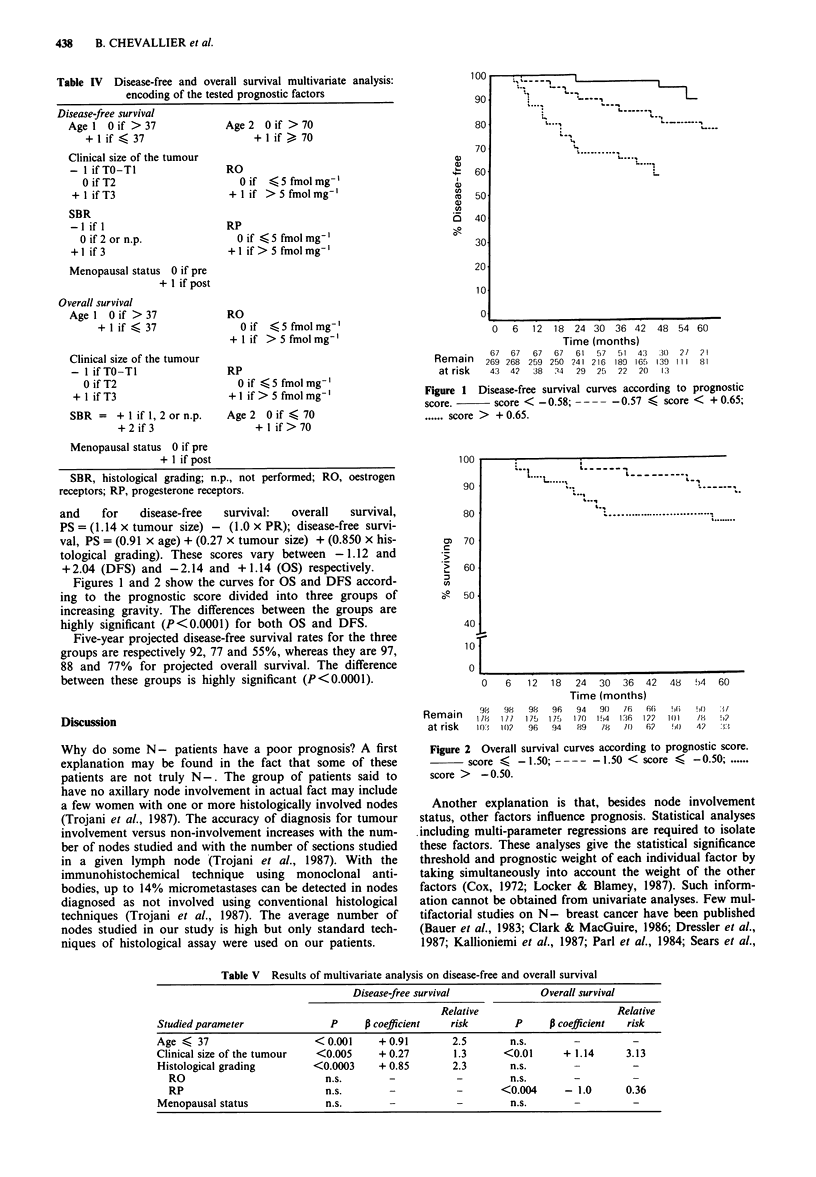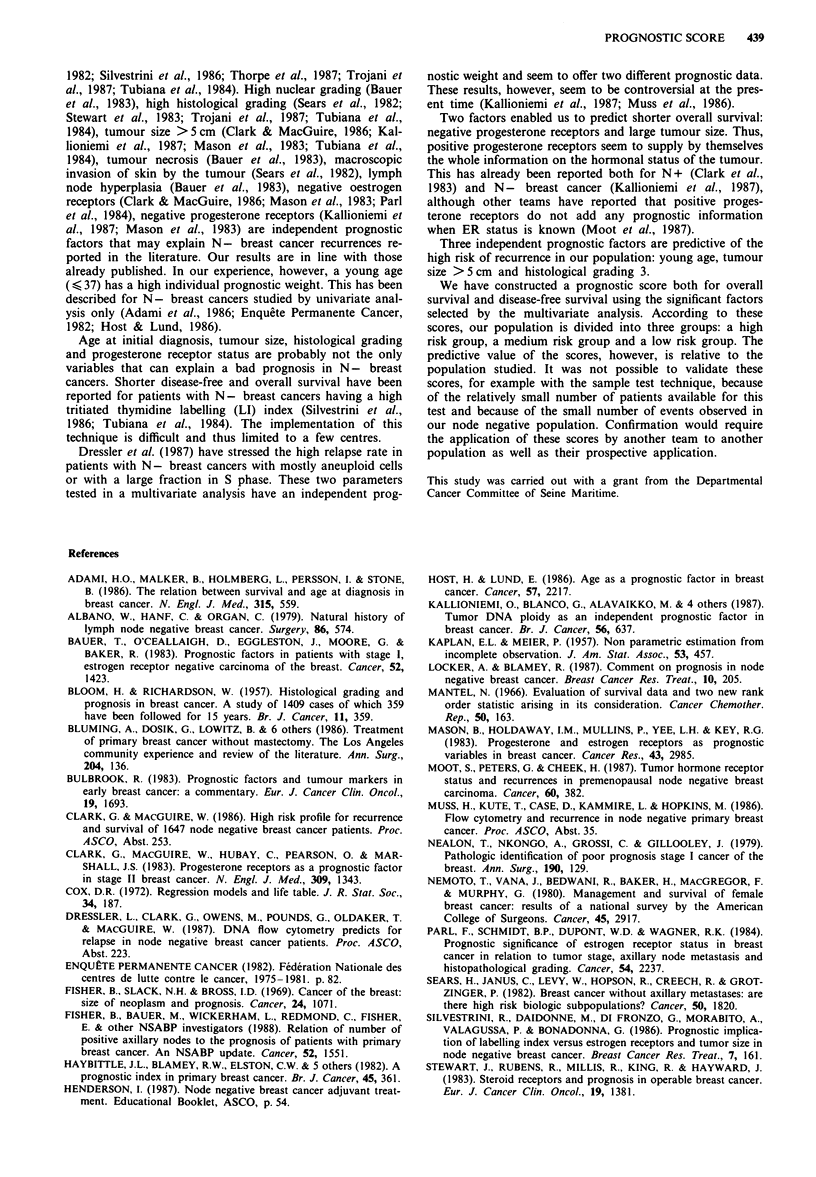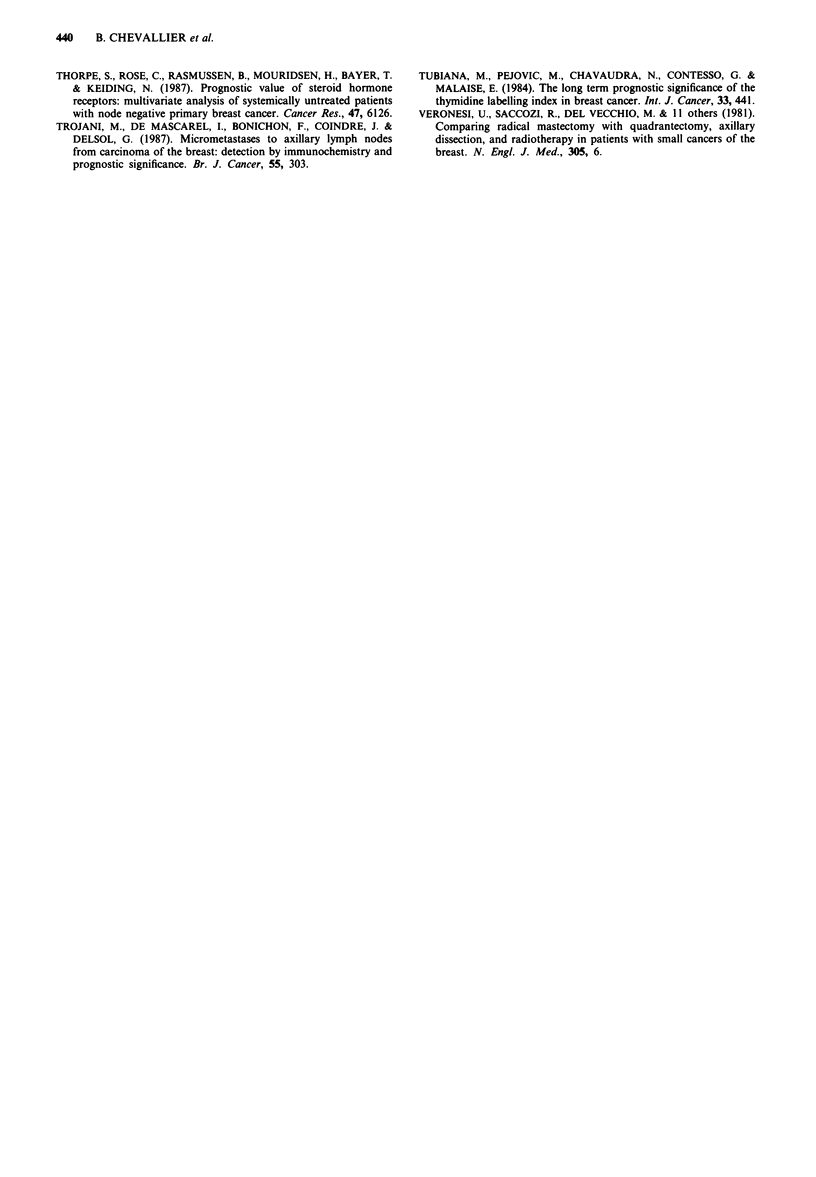# A prognostic score in histological node negative breast cancer.

**DOI:** 10.1038/bjc.1990.96

**Published:** 1990-03

**Authors:** B. Chevallier, V. Mosseri, J. P. Dauce, P. Bastit, J. P. Julien, B. Asselain

**Affiliations:** Service de Médecine Intene et chimiothérapie, Centre H. Becquerel, Rouen, France.

## Abstract

Between October 1977 and December 1983, 379 consecutive patients have been treated for unilateral, non-metastatic breast cancer, either with conservative (n = 205) or radical surgery (n = 174), with axillary dissection in all the cases. None of them had histologically proved lymph node involvement. Oestrogen receptor (ER) and progesterone receptor (PR) levels were measured on each tumour. Levels greater than 5 fmol mg-1 cytosolic protein were considered as positive for both ER and PR. At 5 years, overall survival (OS) and disease-free survival (DFS) are respectively 88% and 78%. Unifactorial analysis using Kaplan and Meier estimates and the log rank test revealed that OS was significantly related to age (P less than 0.05), tumour size (P less than 0.001), histological grading (SBR) (P less than 0.01), ER (P less than 0.001) and PR (P less than 0.001). DFS was significantly related to the same factors. Menopausal status, number of breast tumour foci and previous familial history of breast cancer were not significant. Multifactorial analysis revealed that DFS was significantly related to age (bad prognosis (b.p.) less than or equal to 37 years old), tumour size and histological grading (b.p. SBR = 3), and that OS was significantly related to tumour size and PR (b.p. PR less than or equal to 5 fmol mg-1 protein). A prognostic score has been constructed for both DFS and OS. These scores divide our patients into three significantly different (P less than 0.0001) groups with good, intermediate and bad prognosis.


Br. J. Cancer (1990), 61, 436-440 © Macmillan Press Ltd., 1990

### A prognostic score in histological node negative breast cancer

B. Chevallier[1], V. Mosseri[2], J.P. Dauce[1], P. Bastit[1], J.P. Julien[1] & B. Asselain[2]

[1]*Service de Médecine Interne et chimiothérapie, Centre H. Becquerel, Rue d'Amiens, 76038, Rouen, France; and* [2]*Clinical Research Department, Institut Curie, 26 rue d'Ulm, 75231, Paris, France.*

**Summary** Between October 1977 and December 1983, 379 consecutive patients have been treated for unilateral, non-metastatic breast cancer, either with conservative ($n = 205$) or radical surgery ($n = 174$), with axillary dissection in all the cases. None of them had histologically proved lymph node involvement. Oestrogen receptor (ER) and progesterone receptor (PR) levels were measured on each tumour. Levels >5 fmol $mg^{-1}$ cytosolic protein were considered as positive for both ER and PR. At 5 years, overall survival (OS) and disease-free survival (DFS) are respectively 88% and 78%. Unifactorial analysis using Kaplan and Meier estimates and the log rank test revealed that OS was significantly related to age ($P<0.05$), tumour size ($P<0.001$), histological grading (SBR) ($P<0.01$), ER ($P<0.01$) and PR ($P<0.001$). DFS was significantly related to the same factors. Menopausal status, number of breast tumour foci and previous familial history of breast cancer were not significant. Multifactorial analysis revealed that DFS was significantly related to age (bad prognosis (b.p.) ⩽37 years old), tumour size and histological grading (b.p. SBR = 3), and that OS was significantly related to tumour size and PR (b.p. PR ⩽5 fmol $mg^{-1}$ protein). A prognostic score has been constructed for both DFS and OS. These scores divide our patients into three significantly different ($P<0.0001$) groups with good, intermediate and bad prognosis.

Breast cancer is the most common cause of death from cancer in women. Prognosis is related to different factors including lymph node involvement, hence the better prognosis usually attributed to breast cancer with histologically negative lymph node involvement (N−). For these cancers, however, overall survival and disease-free survival at 5 years range respectively from 73 to 90% and from 60 to 90% (Albano *et al.*, 1979; Bluming *et al.*, 1986; Enquête Permanente Cancer, 1982; Fisher *et al.*, 1969, 1983; Henderson, 1987; Nemoto *et al.*, 1980; Sears *et al.*, 1982; Veronesi *et al.*, 1981). During follow-up, 2–3% N− patients relapse each year (Bulbrook, 1983). A better knowledge of the factors linked with a bad prognosis would help to isolate high risk sub-groups of N− patients for whom randomised trials may be used to establish optimal therapy.

The purpose of this retrospective study was to try and determine such factors indicating a bad prognosis and to construct a prognostic score based on these prognostic factors to isolate a sub-group of high risk N− patients.

### Patients and methods

Between October 1977 and December 1983, 680 consecutive patients were treated at the H. Becquerel Cancer Centre in Rouen for invasive, unilateral, unifocal or multifocal breast cancer. Patients with intraductal carcinoma only were excluded from this study. No visceral or bony metastases were detected on these patients by chest X-ray, bone scintigraphy, hepatic echography or scintigraphy and blood tests. The first therapeutic step was always a surgical operation. Axillary lymph node dissection was performed in every case. An average of 15 nodes per patients (range 2–35) was analysed.

Three hundred and seventy-nine of these patients had no histologically proved lymph node involvement and constitute the basis of this study. The various therapies implemented are detailed in Table I. The average age of the patients at the date of the initial diagnosis was 56 years (range 29–86 years). The main characteristics of the population are detailed in Table II. Surgery was conservative whenever technically possible (size of the tumour compared with breast volume), and this was generally possible for tumours less than 30 mm in diameter, and when the location of the tumour allowed it, although some central tumours have been treated with a tumorectomy. When the treatment was conservative, adjuvant radiotherapy was given using a cobalt-60 source at a dose of 45 Gy to the breast by two opposed fields. A boost of 15 Gy was delivered in the tumoral zone with cobalt-60 in six fractions of 2.5 Gy. No radiotherapy was given on the chest wall if a modified radical mastectomy had been performed. If the tumour was in the inner quadrants, 47.5 Gy adjuvant radiotherapy was given on internal

**Table I** Therapeutic modalities implemented in this study

| *Treatment* | *No adjuvant radiotherapy* | *Adjuvant radiotherapy* | *Total* |
|---|---|---|---|
| Radical modified mastectomy + axillary clearance | 111 | 63 | 174 |
| Quadrantectomy or tumorectomy + axillary clearance | 0 | 205 | 205 |
| Total | 111 | 268 | 379 |

**Table II** Studied parameters and their repartition

| *Studied parameter* | *Number of patients* |
|---|---|
| Age | |
| ⩽37 | 17 |
| 38–70 | 304 |
| >70 | 58 |
| Previous familial history of breast cancer | |
| Mother | 11 |
| Sister | 16 |
| Mother + sister | 3 |
| Menopausal status at initial diagnosis | |
| Non-menopausal | 126 |
| Menopausal | 253 |
| Clinical size of tumour | |
| TO | 1 |
| T1 | 142 |
| T2 | 227 |
| T3 | 9 |
| Histological grading | |
| 1 | 69 |
| 2 | 239 |
| 3 | 38 |
| Not performed | 33 |
| RO | |
| ⩽ 5 fmol $mg^{-1}$ | 139 |
| > 5 fmol $mg^{-1}$ | 240 |
| RP | |
| ⩽ 5 fmol $mg^{-1}$ | 145 |
| > 5 fmol $mg^{-1}$ | 234 |
| Number of tumour foci | |
| 1 | 338 |
| >1 | 41 |

Correspondence: B. Chevallier.
Received 8 June 1989; and in revised form 6 October 1989.

mammary lymph nodes and 45 Gy on the supraclavicular zone. These lymph nodes areas were not irradiated if the tumour was in the outer quadrants. No patient received adjuvant hormonotherapy or chemotherapy.

Oestrogen receptor (ER) and progesterone (PR) concentrations were determined in all cases by single saturation measurement on tumour tissue taken before mastectomy using the dextran coated charcoal method. For both ER and PR, a value over 5 fmol $mg^{-1}$ cytosol protein (fmol $mg^{-1}$) was considered as positive (ER+ or PR+) and a value under or equal to 5 fmol $mg^{-1}$ as negative (ER− or PR−).

The number of intramammary tumour foci was determined by macroscopic examination of the surgical specimen. The histological grading was determined according to the Bloom and Richardson method (1957). It could not be specified in 33 cases because of a specific histological subtype: lobular carcinoma in seven cases, colloid carcinoma in 15 cases and pure comedocarcinoma in 11 cases. The TNM classification (UICC, 1978) was used to express the clinical size of tumours and axillary node status. Menopause was defined as the permanent cessation of the menses for one year or more.

Clinical follow-up consisted of check-ups at 6-month intervals during the first 5 years, then at 1-year intervals. Maximum follow-up time is 110 months and minimum 36 months (median 60 months).

### *Statistical methods*

Univariate analysis was applied to eight parameters likely to influence the recurrence of the tumour (Table II). Age of the patients was divided into the following brackets: <35, 35–40, 41–45 . . . 71–75 and >75. Overall survival and disease free survival were examined for each age bracket. Because there was no statistical difference between each age bracket for patients >37 and <70, for both disease-free and overall survival, we decided to match these patients together and keep only three age-groups: ⩽37, 38–70, >70 years. The effect of these three age groupings does not disappear over time. Histological grading was tested in its whole in both the univariate and multivariate analyses.

Disease-free interval and overall survival (whatever the cause of death may be) were calculated according to the actuarial method of Kaplan and Meier (1957). The significance level of differences between curves (*P*) was determined using the log rank test (Mantel, 1966). Percentages were compared by $\chi^2$ analysis. Later, those factors with a prognostic value in the unifactorial analysis were evaluated using Cox's proportional hazards regression model for censored data with a stepwise procedure (Cox, 1972) in which the hazard of recurrence or death for a given patient is the product of a function of time since mastectomy and a term describing the effects of the prognostic factors. The regression coefficients $\beta 1$, $\beta 2$ etc. were estimated by maximising the partial likelihood function after having encoded each prognostic factor tested in the model. A prognostic score (PS) was calculated for both disease-free and overall survival. The scores use the $\beta$ coefficient shown in Table V and the encoding system described in Table IV. They meet the general equation: $PS = \beta 1.X + \beta 2.Y + \beta 3.Z + ...$ where $\beta 1$, $\beta 2$ and $\beta 3$ are the regression coefficients calculated according to Cox's model and $X$, $Y$ and $Z$ are the significant prognostic variables. Prognosis for a given patient is all the worse as the PS value is high. We then deliberately split these continuous scores into three groups, according to their distribution histogram, and searched for the cut point giving the best discrimination between these groups.

### Results

At 2 and 5 years, overall survival rates are respectively 95.2 ± 2% and 88.6 ± 3.7%, whereas disease-free survival rates are respectively 89.3 ± 3.2% and 77.5 ± 5.0%. Twenty-three cases of local recurrences, three cases of homolateral axillary node recurrences and three cases of loco-regional homolateral recurrences were observed. Two patients developed contralateral breast cancer. Fifty-two patients developed metastatic disease. Out of 40 deaths, 29 were due to cancer evolution. One patient died from an endometrial cancer diagnosed during a routine follow-up examination.

### *Unifactorial analysis*

The results of univariate analysis on disease-free and overall survival are reported in Table III. Age ⩽37, tumour size >5 cm, histological grading SBR 3, oestrogen and/or progesterone receptors ⩽5 fmol $mg^{-1}$ are significantly associated with shorter disease-free or overall survival. Patients aged between 38 and 70 at first diagnosis have the best chance of survival and the longest period free of any relapse. Patients aged ⩽37 at initial diagnosis have the poorest prognosis. Finally, those aged over 70 show a poor overall survival rate but a disease-free survival intermediate between the two groups already mentioned.

Previous familial history of breast cancer, menopausal status and number of tumor foci do not reach the statistical level of significance.

### *Multifactorial analysis*

The five factors found to be significant in the unifactorial analysis plus the factor 'menopausal status' were submitted to the multivariate analysis (Table IV). The results supplied by the model for overall survival and disease-free survival are presented in Table V. Two prognostic parameters are involved in overall survival prediction: tumour size and progesterone receptors. Three parameters are involved in disease-free survival prediction: age at time of initial diagnosis, tumour size and histological grading.

A prognostic score was calculated both for overall survival

**Table III** Univariate analysis of prognostic factors: results on disease-free and overall survival

| *Studied parameter* | *Disease-free survival (P)* | *Overall survival (P)* |
|---|---|---|
| Age (37−; 38–70; 70+) | <0.01 (37−<70+<38–70) | <0.05 (37−<70+<38–70) |
| Previous familial history (yes/no) | n.s. (*P* = 0.86) | n.s. (*P* = 0.92) |
| Menopausal status | n.s. (*P* = 0.50) | n.s. (*P* = 0.17) |
| Clinical size of tumour (T0, T1, T2, T3) | <0.05 (T3<T2<T1–T0) | <0.001 (T3<T2<T1–T0) |
| Number of tumour foci (1; +1) | n.s. (*P* = 0.52) | n.s. (*P* = 0.75) |
| Histological grading (1, 2, 3, (n.p.)) | <0.001 (3<2, n.p.<1) | <0.01 (3<1, 2, n.p.) |
| RO (fmol $mg^{-1}$ −5, +5) | <0.05 (−5<+5) | <0.01 (−5<+5) |
| RP (fmol $mg^{-1}$ −5, +5) | <0.01 (−5<+5) | <0.001 (−5<+5) |

n.s., not significant; n.p. not performed

**Table IV** Disease-free and overall survival multivariate analysis: encoding of the tested prognostic factors

| | |
|---|---|
| *Disease-free survival* | |
| Age 1 0 if > 37<br>+ 1 if ⩽ 37 | Age 2 0 if > 70<br>+ 1 if ⩾ 70 |
| Clinical size of the tumour<br>− 1 if T0−T1<br>0 if T2<br>+ 1 if T3 | RO<br>0 if ⩽5 fmol $mg^{-1}$<br>+ 1 if > 5 fmol $mg^{-1}$ |
| SBR<br>−1 if 1<br>0 if 2 or n.p.<br>+1 if 3 | RP<br>0 if ⩽5 fmol $mg^{-1}$<br>+1 if > 5 fmol $mg^{-1}$ |
| Menopausal status 0 if pre<br>+ 1 if post | |
| *Overall survival* | |
| Age 1 0 if > 37<br>+ 1 if ⩽ 37 | RO<br>0 if ⩽5 fmol $mg^{-1}$<br>+ 1 if > 5 fmol $mg^{-1}$ |
| Clinical size of the tumour<br>− 1 if T0−T1<br>0 if T2<br>+ 1 if T3 | RP<br>0 if ⩽5 fmol $mg^{-1}$<br>+1 if > 5 fmol $mg^{-1}$ |
| SBR = + 1 if 1, 2 or n.p.<br>+ 2 if 3 | Age 2 0 if ⩽ 70<br>+ 1 if > 70 |
| Menopausal status 0 if pre<br>+ 1 if post | |

SBR, histological grading; n.p., not performed; RO, oestrogen receptors; RP, progesterone receptors.

and for disease-free survival: overall survival, PS = (1.14 × tumour size) − (1.0 × PR); disease-free survival, PS = (0.91 × age) + (0.27 × tumour size) + (0.850 × histological grading). These scores vary between −1.12 and +2.04 (DFS) and −2.14 and +1.14 (OS) respectively.

Figures 1 and 2 show the curves for OS and DFS according to the prognostic score divided into three groups of increasing gravity. The differences between the groups are highly significant ($P<0.0001$) for both OS and DFS.

Five-year projected disease-free survival rates for the three groups are respectively 92, 77 and 55%, whereas they are 97, 88 and 77% for projected overall survival. The difference between these groups is highly significant ($P<0.0001$).

### Discussion

Why do some N− patients have a poor prognosis? A first explanation may be found in the fact that some of these patients are not truly N−. The group of patients said to have no axillary node involvement in actual fact may include a few women with one or more histologically involved nodes (Trojani *et al.*, 1987). The accuracy of diagnosis for tumour involvement versus non-involvement increases with the number of nodes studied and with the number of sections studied in a given lymph node (Trojani *et al.*, 1987). With the immunohistochemical technique using monoclonal antibodies, up to 14% micrometastases can be detected in nodes diagnosed as not involved using conventional histological techniques (Trojani *et al.*, 1987). The average number of nodes studied in our study is high but only standard techniques of histological assay were used on our patients.

**Figure 1** Disease-free survival curves according to prognostic score. ——— score < −0.58; ---- −0.57 ⩽ score < +0.65; ...... score > +0.65.

**Figure 2** Overall survival curves according to prognostic score. ——— score ⩽ −1.50; ---- −1.50 < score ⩽ −0.50; ...... score > −0.50.

Another explanation is that, besides node involvement status, other factors influence prognosis. Statistical analyses including multi-parameter regressions are required to isolate these factors. These analyses give the statistical significance threshold and prognostic weight of each individual factor by taking simultaneously into account the weight of the other factors (Cox, 1972; Locker & Blamey, 1987). Such information cannot be obtained from univariate analyses. Few multifactorial studies on N− breast cancer have been published (Bauer *et al.*, 1983; Clark & MacGuire, 1986; Dressler *et al.*, 1987; Kallioniemi *et al.*, 1987; Parl *et al.*, 1984; Sears *et al.*,

**Table V** Results of multivariate analysis on disease-free and overall survival

| | *Disease-free survival* | | | *Overall survival* | | |
|---|---|---|---|---|---|---|
| *Studied parameter* | *P* | *β coefficient* | *Relative risk* | *P* | *β coefficient* | *Relative risk* |
| Age ⩽ 37 | < 0.001 | + 0.91 | 2.5 | n.s. | – | – |
| Clinical size of the tumour | <0.005 | + 0.27 | 1.3 | <0.01 | + 1.14 | 3.13 |
| Histological grading | <0.0003 | + 0.85 | 2.3 | n.s. | – | – |
| RO | n.s. | – | – | n.s. | – | – |
| RP | n.s. | – | – | <0.004 | – 1.0 | 0.36 |
| Menopausal status | n.s. | – | – | n.s. | – | – |

1982; Silvestrini *et al.*, 1986; Thorpe *et al.*, 1987; Trojani *et al.*, 1987; Tubiana *et al.*, 1984). High nuclear grading (Bauer *et al.*, 1983), high histological grading (Sears *et al.*, 1982; Stewart *et al.*, 1983; Trojani *et al.*, 1987; Tubiana *et al.*, 1984), tumour size $>5$ cm (Clark & MacGuire, 1986; Kallioniemi *et al.*, 1987; Mason *et al.*, 1983; Tubiana *et al.*, 1984), tumour necrosis (Bauer *et al.*, 1983), macroscopic invasion of skin by the tumour (Sears *et al.*, 1982), lymph node hyperplasia (Bauer *et al.*, 1983), negative oestrogen receptors (Clark & MacGuire, 1986; Mason *et al.*, 1983; Parl *et al.*, 1984), negative progesterone receptors (Kallioniemi *et al.*, 1987; Mason *et al.*, 1983) are independent prognostic factors that may explain N− breast cancer recurrences reported in the literature. Our results are in line with those already published. In our experience, however, a young age ($\leqslant 37$) has a high individual prognostic weight. This has been described for N− breast cancers studied by univariate analysis only (Adami *et al.*, 1986; Enquête Permanente Cancer, 1982; Host & Lund, 1986).

Age at initial diagnosis, tumour size, histological grading and progesterone receptor status are probably not the only variables that can explain a bad prognosis in N− breast cancers. Shorter disease-free and overall survival have been reported for patients with N− breast cancers having a high tritiated thymidine labelling (LI) index (Silvestrini *et al.*, 1986; Tubiana *et al.*, 1984). The implementation of this technique is difficult and thus limited to a few centres.

Dressler *et al.* (1987) have stressed the high relapse rate in patients with N− breast cancers with mostly aneuploid cells or with a large fraction in S phase. These two parameters tested in a multivariate analysis have an independent prognostic weight and seem to offer two different prognostic data. These results, however, seem to be controversial at the present time (Kallioniemi *et al.*, 1987; Muss *et al.*, 1986).

Two factors enabled us to predict shorter overall survival: negative progesterone receptors and large tumour size. Thus, positive progesterone receptors seem to supply by themselves the whole information on the hormonal status of the tumour. This has already been reported both for N+ (Clark *et al.*, 1983) and N− breast cancer (Kallioniemi *et al.*, 1987), although other teams have reported that positive progesterone receptors do not add any prognostic information when ER status is known (Moot *et al.*, 1987).

Three independent prognostic factors are predictive of the high risk of recurrence in our population: young age, tumour size $>5$ cm and histological grading 3.

We have constructed a prognostic score both for overall survival and disease-free survival using the significant factors selected by the multivariate analysis. According to these scores, our population is divided into three groups: a high risk group, a medium risk group and a low risk group. The predictive value of the scores, however, is relative to the population studied. It was not possible to validate these scores, for example with the sample test technique, because of the relatively small number of patients available for this test and because of the small number of events observed in our node negative population. Confirmation would require the application of these scores by another team to another population as well as their prospective application.

This study was carried out with a grant from the Departmental Cancer Committee of Seine Maritime.

### References

ADAMI, H.O., MALKER, B., HOLMBERG, L., PERSSON, I. & STONE, B. (1986). The relation between survival and age at diagnosis in breast cancer. *N. Engl. J. Med.*, **315**, 559.

ALBANO, W., HANF, C. & ORGAN, C. (1979). Natural history of lymph node negative breast cancer. *Surgery*, **86**, 574.

BAUER, T., O'CEALLAIGH, D., EGGLESTON, J., MOORE, G. & BAKER, R. (1983). Prognostic factors in patients with stage I, estrogen receptor negative carcinoma of the breast. *Cancer*, **52**, 1423.

BLOOM, H. & RICHARDSON, W. (1957). Histological grading and prognosis in breast cancer. A study of 1409 cases of which 359 have been followed for 15 years. *Br. J. Cancer*, **11**, 359.

BLUMING, A., DOSIK, G., LOWITZ, B. & 6 others (1986). Treatment of primary breast cancer without mastectomy. The Los Angeles community experience and review of the literature. *Ann. Surg.*, **204**, 136.

BULBROOK, R. (1983). Prognostic factors and tumour markers in early breast cancer: a commentary. *Eur. J. Cancer Clin. Oncol.*, **19**, 1693.

CLARK, G. & MACGUIRE, W. (1986). High risk profile for recurrence and survival of 1647 node negative breast cancer patients. *Proc. ASCO*, Abst. 253.

CLARK, G., MACGUIRE, W., HUBAY, C., PEARSON, O. & MARSHALL, J.S. (1983). Progesterone receptors as a prognostic factor in stage II breast cancer. *N. Engl. J. Med.*, **309**, 1343.

COX, D.R. (1972). Regression models and life table. *J. R. Stat. Soc.*, **34**, 187.

DRESSLER, L., CLARK, G., OWENS, M., POUNDS, G., OLDAKER, T. & MACGUIRE, W. (1987). DNA flow cytometry predicts for relapse in node negative breast cancer patients. *Proc. ASCO*, Abst. 223.

ENQUÊTE PERMANENTE CANCER (1982). Fédération Nationale des centres de lutte contre le cancer, 1975–1981. p. 82.

FISHER, B., SLACK, N.H. & BROSS, I.D. (1969). Cancer of the breast: size of neoplasm and prognosis. *Cancer*, **24**, 1071.

FISHER, B., BAUER, M., WICKERHAM, L., REDMOND, C., FISHER, E. & other NSABP investigators (1988). Relation of number of positive axillary nodes to the prognosis of patients with primary breast cancer. An NSABP update. *Cancer*, **52**, 1551.

HAYBITTLE, J.L., BLAMEY, R.W., ELSTON, C.W. & 5 others (1982). A prognostic index in primary breast cancer. *Br. J. Cancer*, **45**, 361.

HENDERSON, I. (1987). Node negative breast cancer adjuvant treatment. Educational Booklet, ASCO, p. 54.

HOST, H. & LUND, E. (1986). Age as a prognostic factor in breast cancer. *Cancer*, **57**, 2217.

KALLIONIEMI, O., BLANCO, G., ALAVAIKKO, M. & 4 others (1987). Tumor DNA ploidy as an independent prognostic factor in breast cancer. *Br. J. Cancer*, **56**, 637.

KAPLAN, E.L. & MEIER, P. (1957). Non parametric estimation from incomplete observation. *J. Am. Stat. Assoc.*, **53**, 457.

LOCKER, A. & BLAMEY, R. (1987). Comment on prognosis in node negative breast cancer. *Breast Cancer Res. Treat.*, **10**, 205.

MANTEL, N. (1966). Evaluation of survival data and two new rank order statistic arising in its consideration. *Cancer Chemother. Rep.*, **50**, 163.

MASON, B., HOLDAWAY, I.M., MULLINS, P., YEE, L.H. & KEY, R.G. (1983). Progesterone and estrogen receptors as prognostic variables in breast cancer. *Cancer Res.*, **43**, 2985.

MOOT, S., PETERS, G. & CHEEK, H. (1987). Tumor hormone receptor status and recurrences in premenopausal node negative breast carcinoma. *Cancer*, **60**, 382.

MUSS, H., KUTE, T., CASE, D., KAMMIRE, L. & HOPKINS, M. (1986). Flow cytometry and recurrence in node negative primary breast cancer. *Proc. ASCO*, Abst. 35.

NEALON, T., NKONGO, A., GROSSI, C. & GILLOOLEY, J. (1979). Pathologic identification of poor prognosis stage I cancer of the breast. *Ann. Surg.*, **190**, 129.

NEMOTO, T., VANA, J., BEDWANI, R., BAKER, H., MACGREGOR, F. & MURPHY, G. (1980). Management and survival of female breast cancer: results of a national survey by the American College of Surgeons. *Cancer*, **45**, 2917.

PARL, F., SCHMIDT, B.P., DUPONT, W.D. & WAGNER, R.K. (1984). Prognostic significance of estrogen receptor status in breast cancer in relation to tumor stage, axillary node metastasis and histopathological grading. *Cancer*, **54**, 2237.

SEARS, H., JANUS, C., LEVY, W., HOPSON, R., CREECH, R. & GROTZINGER, P. (1982). Breast cancer without axillary metastases: are there high risk biologic subpopulations? *Cancer*, **50**, 1820.

SILVESTRINI, R., DAIDONNE, M., DI FRONZO, G., MORABITO, A., VALAGUSSA, P. & BONADONNA, G. (1986). Prognostic implication of labelling index versus estrogen receptors and tumor size in node negative breast cancer. *Breast Cancer Res. Treat.*, **7**, 161.

STEWART, J., RUBENS, R., MILLIS, R., KING, R. & HAYWARD, J. (1983). Steroid receptors and prognosis in operable breast cancer. *Eur. J. Cancer Clin. Oncol.*, **19**, 1381.

THORPE, S., ROSE, C., RASMUSSEN, B., MOURIDSEN, H., BAYER, T. & KEIDING, N. (1987). Prognostic value of steroid hormone receptors: multivariate analysis of systemically untreated patients with node negative primary breast cancer. *Cancer Res.*, **47**, 6126.

TROJANI, M., DE MASCAREL, I., BONICHON, F., COINDRE, J. & DELSOL, G. (1987). Micrometastases to axillary lymph nodes from carcinoma of the breast: detection by immunochemistry and prognostic significance. *Br. J. Cancer*, **55**, 303.

TUBIANA, M., PEJOVIC, M., CHAVAUDRA, N., CONTESSO, G. & MALAISE, E. (1984). The long term prognostic significance of the thymidine labelling index in breast cancer. *Int. J. Cancer*, **33**, 441.

VERONESI, U., SACCOZI, R., DEL VECCHIO, M. & 11 others (1981). Comparing radical mastectomy with quadrantectomy, axillary dissection, and radiotherapy in patients with small cancers of the breast. *N. Engl. J. Med.*, **305**, 6.